# Super-strong and high-performance electrical film heater derived from silver nanowire/aligned bacterial cellulose film

**DOI:** 10.1186/s40643-023-00669-w

**Published:** 2023-08-23

**Authors:** Guichun Hu, Amir Varamesh, Na Zhong, Fangong Kong, Jinguang Hu

**Affiliations:** 1 Department of Chemical & Petroleum Engineering, Schulich School of Engineering, Calgary, AB T2N 1N4 Canada; 2 State Key Laboratory of Biobased Material and Green Papermaking, Faculty of Light Industry, Shandong Academy of Sciences, Qilu University of Technology, Jinan, 250353 People’s Republic of China

**Keywords:** Bacterial cellulose film, Silver nanowire, Joule heater, Mechanical strength, Flexibility

## Abstract

High-performance electrical Joule heaters with high mechanical properties, low driving voltage, rapid response, and flexibility are highly desirable for portable thermal management. Herein, by using aligned bacterial cellulose (BC) and silver nanowire (AgNW), we fabricated a novel film heater based on Joule heating phenomena. The aligned BC film prepared by stretching BC hydrogel and hot-pressing drying technology showed outstanding mechanical properties and flexibility. The ultrahigh strength of up to 1018 MPa and the toughness of 20 MJ/m^3^ were obtained for the aligned BC film with 40% wet-stretching (BC-40). In addition, the aligned BC film could be folded into desirable shapes. The AgNW was spray-coated on the surface of aligned BC-40 film and then covered with polydimethylsiloxane to form a P@AgNW@BC heater. P@AgNW@BC heater showed excellent conductivity, which endowed the film heater with an outstanding Joule heating performance. P@AgNW@BC heater could reach ~ 98 ℃ at a very low driving voltage of 4 V with a rapid heating response (13 s) and long-term temperature stability. The P@AgNW@BC heater with such an outstanding heating performance can be used as a flexible heating device for different applications in daily life like deicing/defogging device, wearable thermotherapy, etc.Affiliations: Please check and confirm that the authors and their respective affiliations have been correctly identified and amend if necessary.yes, we confirmed the affiliations are correct. Article title: Kindly check and confirm the edit made in the article title.Thanks, the title is no problem.

**Graphical Abstract:**

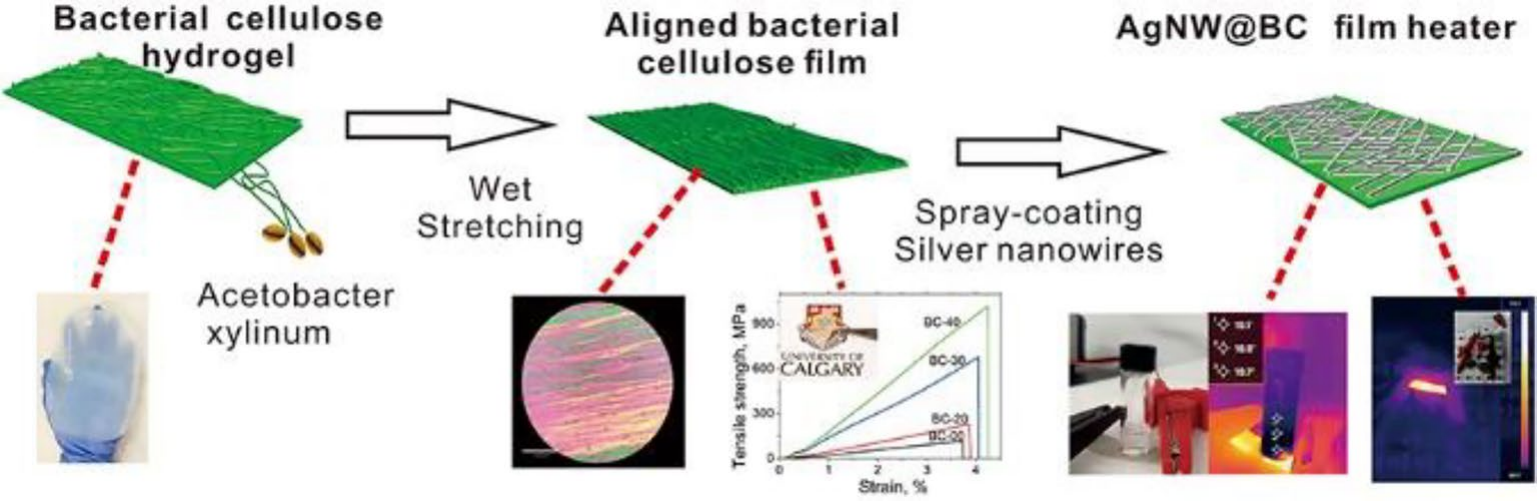

**Supplementary Information:**

The online version contains supplementary material available at 10.1186/s40643-023-00669-w.

## Introduction

Electrical heaters based on the Joule heating effect have attracted significant attention from both academia and industry (Hossain et al. 2021; Veeramuthu et al. 2019). The high efficiency of electrical heaters in converting electricity into heat has led to widespread use of them for a variety of purposes in daily life, including snow-removal devices, local heating, deforesting and defogging, personal thermal management, healthcare thermotherapy, etc. (Meng et al. 2019). In light of the fast-growing and emerging need for electrical heaters, it is extremely important to develop electrical film heaters that fulfill the end-users demanding specifications. These include excellent mechanical properties, being flexible and shapable, having low driving voltage, satisfying heating temperature, rapid response, outstanding heating reliability, durability, and portability (Hossain et al. 2021; Wang et al. 2020). To achieve the above-mentioned characteristics, electrical heaters must have a well-designed structure.We have ignored to process the Graphical abstract caption Kindly check and confirm and amend if necessary.Thanks, the graphical abstract is correct.

Typically, electrical film heaters are fabricated by sputtering, coating, or printing conductive materials onto flexible substrates (Wu et al. 2022; Jyothibasu et al. 2019). Various conductive materials, such as polymers, metal nanowires, and carbon-based materials have been widely studied over the past several decades for fabrication of the film heaters (Yu et al. 2019; Zhang et al. 2018; Jia et al. 2018). Among these materials, silver nanowire (AgNW), has attracted considerable attention owing to its unique features, including high conductivity, considerable flexibility, and processability in aqueous dispersions (Ma et al. 2019; Zhou et al. 2020a, b). For example, Cheng et al. fabricated a cellulose nanofiber/silver nanowire nano paper electrical heater with an excellent heating temperature (~ 220 °C) (Cheng et al. 2022). As for the substrates in electrical heaters, many flexible membrane materials, such as petroleum plastic, paper, and natural polymer films have been used (Cao et al. 2019, 2020). Among various natural polymer films, cellulose film possesses many unique advantages, such as nontoxicity, biocompatibility, flexibility, and sustainability, which makes it a widely available and ideal candidate substrate for electrical film heaters (Kandhola et al. 2020; Song et al. 2019; Vallejo et al. 2021; Zhou et al. 2021;). Liang et al. fabricated a regenerated cellulose/silver nanowire composite film heater which achieves 107 ℃ at a voltage of 2 V and tensile strength of 61 MPa (Liang et al. 2020). Lu et al. prepared the cellulose/multiwalled carbon nanotube composite film with high Joule heating performance to reach 166 ℃ at the input voltage of 2.0 V and low tensile strength of 110 MPa (Lu et al. 2022). Zhou et al. fabricated cellulose nanofiber/MXene film with an outstanding and steady Joule heating performance to achieve more than 100 ℃ at 6 V voltage and mechanical strength of 112.5 MPa (Zhou et al. 2020a, b). These cellulose-based electrical heaters exhibit excellent Joule heating performances, but their mechanical properties are low and unsatisfying, which limits the application of cellulose-based electrical heaters.Author details: As per journal standard requirements Email address was necessary for corresponding authors. Upon checking Email address for the corresponding author “Jinguang Hu” was not provided in the manuscript. In this regard, Please be informed that the Email address was taken from the submission system. Kindly check and confirmThanks, the Dr. Jinguang Hu's email address provided by the submission system is correct. 

Aerobic bacteria produces bacterial cellulose (BC) with randomly oriented fibrous network structure under static immersion. As compared with plant-based cellulose, BC fibers possess high specific surface area/purify/crystallinity/degree of polymerization, along with better biocompatibility (Zikmanis et al. 2021). In addition, the align BC film prepared by wet-stretching method shows excellent mechanical properties (Wang et al. 2018). In this work, we report a novel ultratough, flexible, and high-performance electrical heater derived from super-strong aligned BC film and highly conductive AgNW via a facile and efficient spray-coating method. The aligned BC film prepared by the wet-stretching and hot-pressing drying process exhibited high-record strength (1018 MPa) and excellent toughness (20 MJ m^−3^). The flexible and ultrastrong aligned BC film acted as high-performance substrates and was spray-coated with AgNW to form AgNW@BC composite film. To avoid the AgNW oxidization, the AgNW@BC composite film was coated with polydimethylsiloxane (PDMS) to fabricate the P@AgNW@BC film heater. The P@AgNW@BC electrical film heater not only demonstrated superior mechanical robustness and flexibility, but also an outstanding Joule heating performance (up to at 98 ℃ at 4 V). The designed P@AgNW@BC electrical film is a promising candidate for applications in flexible electrical heaters in diverse areas.

## Experimental

### Materials

The BC was self-made at the in-house lab (University of Calgary, Canada). The AgNO_3_, poly (N-vinyl-2-pyrrolidone) (PVP, molecular weight of 15,000), methanol, ethanediol (EG), glucose, yeast extract, peptone, sodium phosphate dibasic, citric acid, magnesium sulfate, NaCl and NaOH of analytical grade, polydimethylsiloxane (PDMS) and plasticizer were bought from Sigma Aldrich (Canada). *Acetobacter xylinum* (ATCC 23769) was purchased from American Type Culture Collection (USA). *Escherichia coli* (*E. coli,* ATCC 25922) was purchased from the American Culture Collection. All the chemicals were used as received without further purification.Article structure: Kindly check whether the section headings have been identified correctly and amend if any.Thanks, the section headings are correctly identified.

### BC films fabrication

*Acetobacter xylinum* was incubated with HS-medium (glucose 20 g/L, yeast extract 5 g/L, bacto-peptone 5 g/L, sodium phosphate dibasic 2.7 g/L, citric acid 1.15 g/L, and magnesium sulphate 1.0 g/L) for 21 days in a static culture. Then BC membranes were boiled in 0.1 wt % NaOH for 60 min to get rid of the remaining HS-medium and microorganisms, and finally repeatedly rinsed with pure water until the filtrate became neutral.

The purified BC films were cut into a rectangular shape and measured its length which marked as the original length. Then the BC films were wet-drawn using a tensile tester (Series 1500, Qualitest) at a speed of 1 mm/min to reach a strain of 10%. Subsequently, the 10% wet-drawn BC films were unloaded from the tensile tester, shaken repeatedly, and further stretched to achieve a higher wet-drawing strain of 20%. Then, it has been unloaded and the last step was repeated to reach the wet-drawn strain of 30% and 40%. Finally, the BC film samples were press-dried at 60 ℃ for 24 h with the hot press machine (heat press machine, mg-h 10 t 35 v, DABPRSS Technologies, China). The range of wet-drawn strain (0%, 20%, 30%, and 40%) was selected based on (Wang et al. 2018). For comparison purpose, the pristine BC film (0%) was also prepared by hot-press drying at 60 ℃ for 24 h. The BC films with wet-drawn strain of 0%, 20%, 30%, and 40% were named BC-00, BC-20, BC-30, and BC-40, respectively.

### Preparation of P@AgNW@BC composite film

Briefly, 60 ml of PVP/EG solution (0.18 mol/L) and 30 µL of NaCl aqueous solution (0.1 mol/L) were added into a flask and preheated at 150 ℃ in an oil bath for 60 min under stirring. Then, 30 ml of freshly prepared AgNO_3_/EG solution (0.12 mol/l) was added dropwise to the flask under stirring and heated at 150 ℃ for another 240 min. After the reaction, the solution was cooled down at room temperature, then a large amount of methanol was added and the mixture was centrifuged (10,000 rpm/min, 10 min) six times to purify the silver nanowires (AgNWs). Finally, AgNWs were redispersed in methanol for the following application.

The dispersed AgNWs solution (10 mg/ml, 3 ml) was spray-coated onto the aligned BC-40 film (area size ~ 15 cm^2^) using a Paasche airbrush (VL Siphon feed, 0.73 mm nozzle internal diameter, Paasche airbrush, USA), maintaining ~ 7 cm distance from spray nozzle to film and air bar at 1.6 bar. Then, the spray-coated aligned BC film (AgNW@BC) was successively dried under ambient conditions (10 min) and in 110 ℃ oven for 10 min. This process was, respectively, repeated 2, 3, 5, and 7 times to obtain AgNW@BC composite film with 2, 3, 5, and 7 AgNW layers. Finally, the AgNW@BC composite films were spin-coated with PDMS to obtain P@AgNW@BC composite film. The AgNW layers of P@AgNW@BC composite films varied in 2, 3, 5, and 7 were named P@AgNW@BC-2, P@AgNW@BC-3, P@AgNW@BC-5, and P@AgNW@BC-7, respectively.

### Characterization

The morphological features of the samples were examined using a field emission scanning electron microscope (SEM, JSM-6700F, JEOL, Japan). The wide-angle X-ray diffractogram (XRD) was acquired on an X-ray diffractometer (D/max-2500, Rigaku Denki, Japan) at the scanning speed of 2°/min. The Olympus BH-2 optical microscope was used to observe the aligned BC films with the assistance of two polarized light filters. Mechanical performances of the original and wet-stretching BC films in the path of the wet-stretching were investigated on the tensile tester (Series 1500, Qualitest) at a speed of 1 mm/min and each samples tested 3 times. Young’s modulus was acquired as the slope at low strain and toughness was determined as the area under the stress–strain curve. Light transmittance of the samples was measured using a visible spectrophotometer (CARY 50 Bio, Varian) with a wavelength range of 380 to 730 nm. The sheet resistance was measured using a four-point probe tester (2258C, Suzhou Lattice Electronics Co., Ltd., China). The electric heating performance of the sample was characterized under a constant applied voltage of 1–7 V and current of 2 A with an infrared camera (FLIR A300, FLIR Systems, USA) and sourcemeter (2400, Keithley, USA).

### Antibacterial testing

The antimicrobial activities of P@AgNW@BC films were analyzed by inhibition zone testing. *Escherichia coli* (*E. coli*) is a Gram-negative, rod-shaped bacterium found in lower intestine of warm blooded organisms (endotherms). Due to its strong ability to survive in nature, it also can be found in skin and is often used to measure the antibacterial properties of electrical heater (Gupta et al. 2013; Zhu et al. 2022; Du et al. 2022) So, *E. coli* was used to test the antibacterial activity of P@AgNW@BC films. *E. coli* was cultivated in 10 ml Mueller–Hinton Broth (Cation adjusted) at 37 ℃ for 12 h. Then, 150 µl *E. coli* suspension (4.5 × 10^6^ CFU/ml) was uniformly spread over a Luria–Bertani agar plate. Samples were carefully placed on the center of the agar plate and incubated in a constant temperature incubator at 37 ℃ for 24 h. The image of the suppression area was taken by a ChemiDoc Imaging System (Bio-RAD ChemiDocTM MP Imaging System, UK).

## Results and discussion

### Fabrication strategy and structural characterization of aligned BC film

**Fig. 1:**
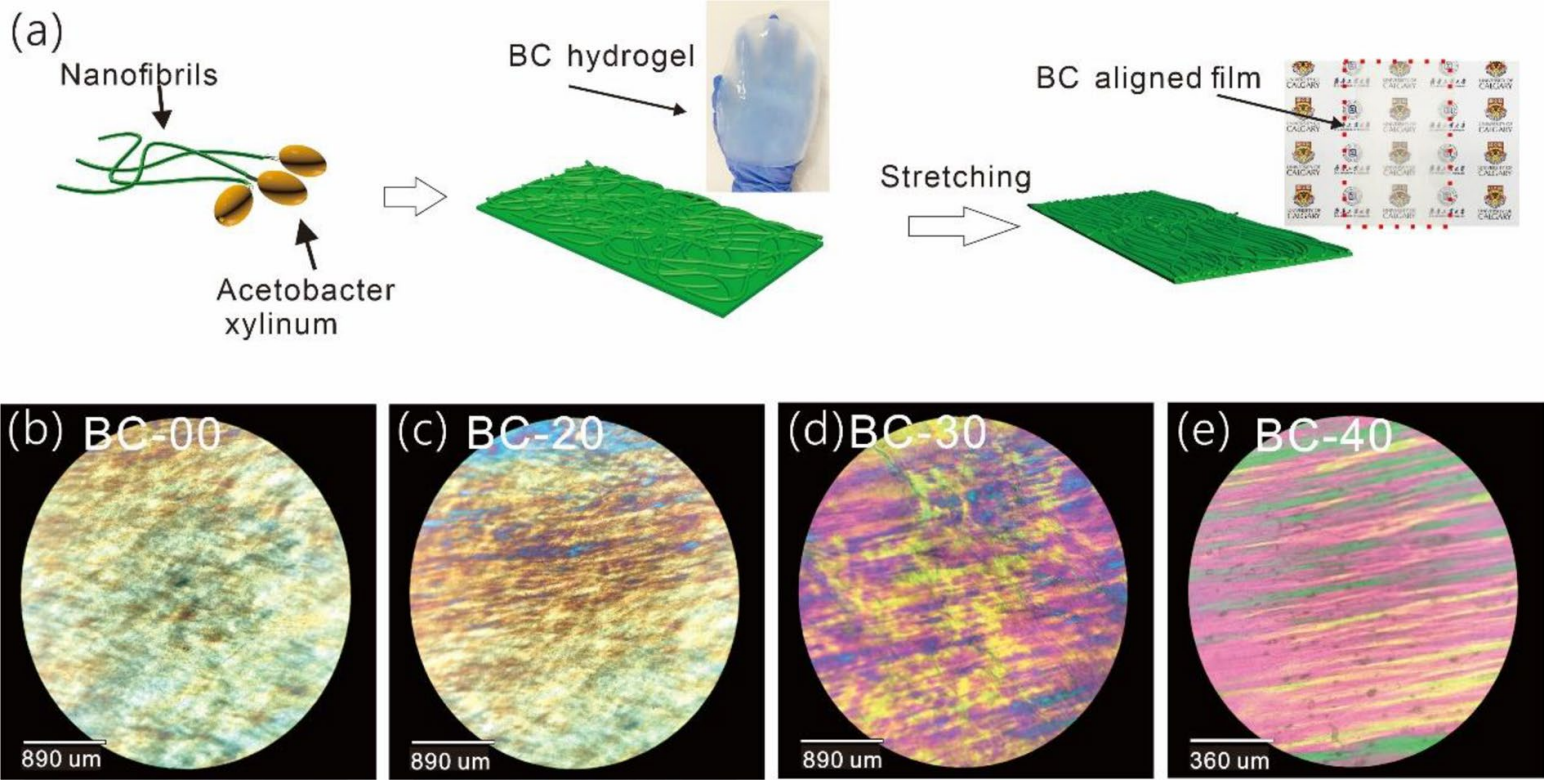
**a** The schematic illustration of wet-stretching BC film fabrication; **b–e** polarized optical microscopy image of BC films with different wet-drawing **b** BC-00, **c** BC-20, **d** BC-30, and **e** BC-40

The pristine BC nanofibrils extruded by *Acetobacter xylinum* are randomly distributed in a wet membrane and then the wet nanofibril membrane was stretched to form BC film with the aligned cellulose nanofibrils (shown in Fig. 1a). BC film's wet-stretching percentage is defined as the ratio between the value of the stretching-induced length and the original length. The polarized optical microscopy images of the BC film with different wet-stretching percentages are shown in Fig. 1b–e. The pristine BC film (BC-00) exhibited randomly distributed brightly colored regions. However, colorful discontinuous strips were observed in the BC film with 20% wet-stretching (BC-20) indicating elongation of the cellulose fibril crystalline domains with the wet-stretching direction. As the wet-stretching percentage increased to 30% (BC-30), the colorful strips became more pronounced. This progression of colours was consistent with increasing alignment, as shown in the BC film with a 40% wet-stretching percentage (BC-40).

**Fig. 2:**
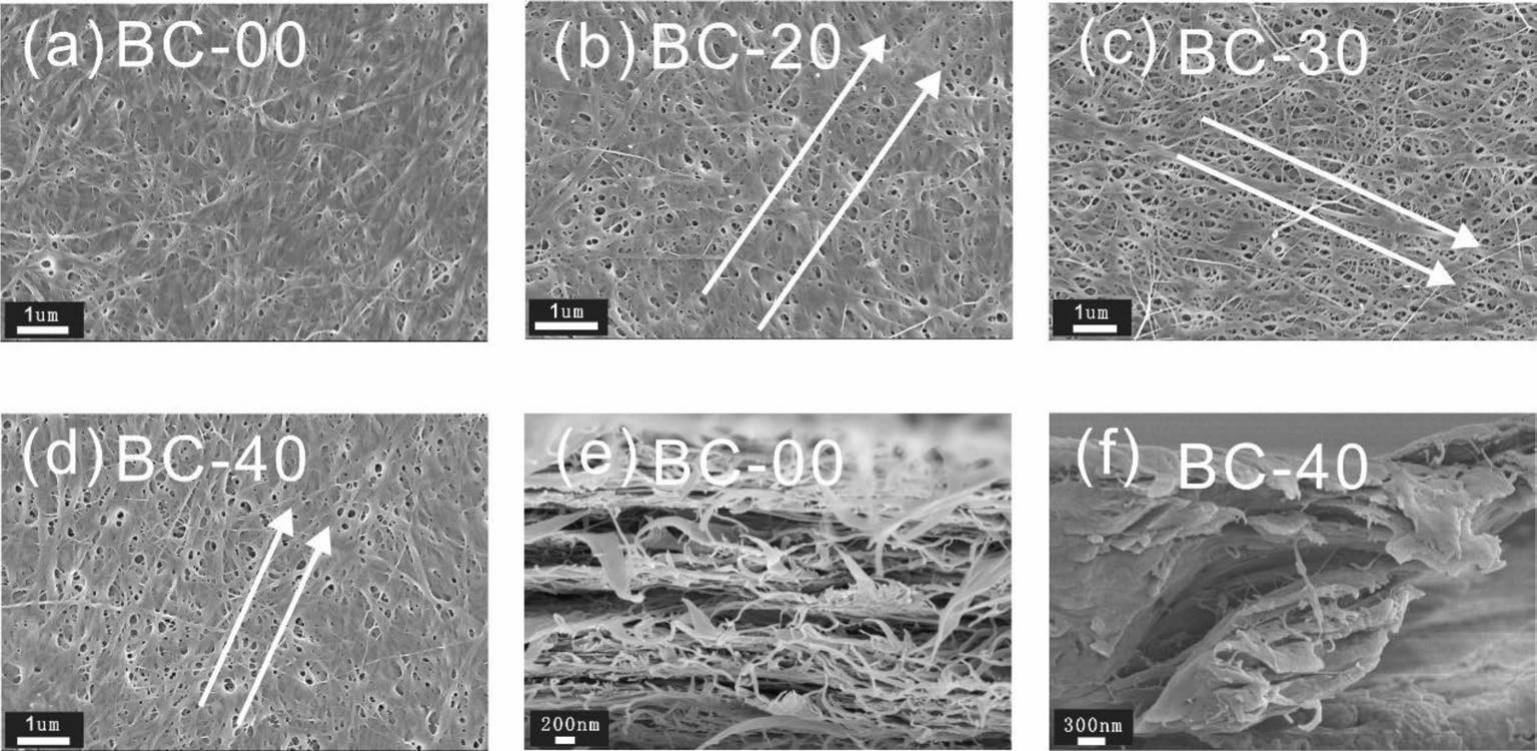
SEM images of the surface of **a** BC-00, **b** BC-20, **c** BC-30, and **d** BC-40 wet-stretching BC films; **e–f** cross-sectional images of **e** BC-00 film and **f** BC-40 film

The surface morphologies of BC films with different wet-stretching percentages were also investigated by field emission scanning electron microscope (FE-SEM) as shown in Fig. 2. The original BC-00 film contains randomly distributed nanofibrils, while the BC-20 film showed an increasing amount of aligned nanofibrils. After further increasing the wet-stretching percentage to 30%, more aligned nanofibrils of BC-30 were observed. For the BC-40 film, there were few wrinkles parallel to each other, showing a well-aligned structure on a microscale. In the cross-section morphology of the pristine BC-00, a layered structure was apparent in the pristine BC-00 film. However, the cross-section morphology of the BC-40 film showed some obvious fibril bundles, which was also observed in accordance with the literature results (Wang et al. 2018). This observation is assumed to be related to the formation of strong hydrogen bonding between the highly aligned nanofibrils (Wu et al. 2020; Wang et al. 2018).

**Fig. 3:**
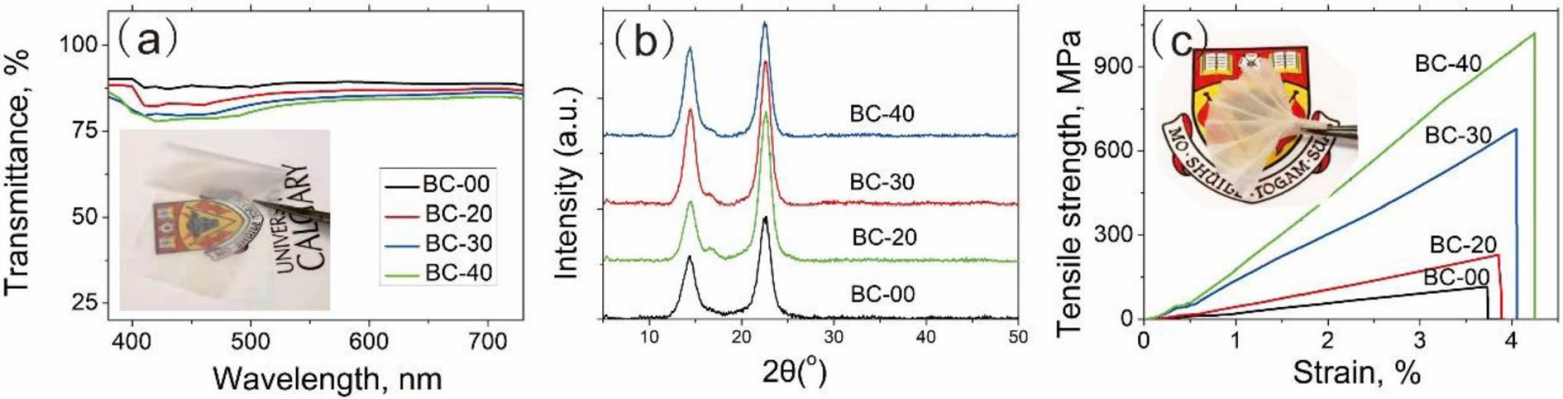
**a** Transmittance, **b** XRD and **c** tensile strength of BC films with different wet-stretching

The light transmittances of the different BC films are depicted in Fig. 3a. Both the original and stretched films show high transmittance covering the entire visible wavelength ranging from 380 to 730 nm. The light transmittance slightly decreased with the increase of the wet-stretching percentage of BC films. The transmittances of the BC films were incredibly high with transmittances of above 88% at 550 nm. The University of Calgary logo covered with BC-40 film could be clearly observed in Fig. 3a, which further indicated the high transmittance of BC films.

The microstructural changes of BC films were investigated by XRD (Fig. 3b). BC films with different wet-stretching percentages showed significant diffraction peaks at 14.6°, 16.3° and 22.5° with a slightly distinguishable difference in their intensities. The peaks were indexed to the (100), (010) and (110) crystallographic planes of cellulose Iα, respectively, as reported in the previous studies (Zikmanis et al. 2021). BC-40 and BC-30 showed higher intensities at the (100) and (110) angles, proving that they pose highly crystalline structures. From their crystallinity and the wet-stretching percentage, it can be concluded that the wet-stretching improves the crystallization of BC film.

The tensile strength and toughness of BC films that undergo different stretching levels were tested (Fig. 3c, Table 1). Interestingly, a higher wet-stretching strain level led to an increase in strength and modulus along with improved toughness. The tensile strength of the original BC-00 film was 116.5 MPa. When it stretched to 20%, the aligned BC-20 film showed a significantly improved tensile strength (229.9 MPa) as well as toughness (4.0 MJ/m), which were 1.9 times and 2.0 times higher than the original one, respectively. The exceptional mechanical properties of the BC films were associated with highly crystalline cellulose from bacterial biosynthesis (Wu et al. 2020; Wang et al. 2018). The mechanical properties of the BC film were further enhanced with increasing structural alignment. As the wet-stretching strain increased to 40%, the tensile strength reached 1018.8 MPa, and Young’s modulus increased to 20.2 MJ/m^3^. The increase in Young’s modulus indicates the reduction of the cellulose film deformation. The reason for the superb mechanical properties of our BC film is due to the wet stretching-induced nanoscale alignment of the nanofibrils and densely packed microfibril bundles. As shown SEM images (Fig. 2), the BC film fabricated after high wet-stretching percentage (e.g., BC-40) showed a well-aligned nanofibril structure. According to FTIR spectrograms (Additional file 1: Fig. S1), the characteristic vibrational modes of BC films after different wet-stretching percentages were almost same in the typical fingerprint regions as reported earlier (Daya et al. 2013; Shibayama et al. 1990). The characteristic peaks located at 3500–3100, 1430, 1111, 1046, and 892 cm-1 were assigned to O–H, CH_2_, C–C, C–O–H, and C–O–C groups, respectively. (Daya et al. 2013) have reported that signals near 892 and 1430 are designated as an “amorphous” band and “crystallinity” band, respectively. The intensities of BC-30-BC-40 at 1430 cm^−1^ obviously increased compared with BC-00. The intensities of BC films at 892 cm^−1^ are almost no change. This phenomenon is coincided with the results of XRD of BC films indicating the improvement of crystallization of BC film after the wet-stretching. In addition, the densities of BC films (Additional file 1: Fig. S2) enhanced with the increase of wet-stretch percentage, indicating the strong interaction among the cellulose nanofibers. Similar results have also been observed and studied by others (Wang et al. 2018; Wu et al. 2020). For example, Wang et al. (Wang et al. 2018) investigated the underlying mechanism of the superb mechanical properties of the highly aligned BC film with molecular dynamics simulations and found that the well-aligned model exhibits both strength and toughness that are exceptionally higher than those of the randomly aligned model. In addition, the wet-stretching BC-40 film was extremely foldable and could be folded into a paper fan (Fig. 3c). Upon unfolding, the BC film did not show visible degradation and the tensile strengths of BC films after folding 100 times (Additional file 1: Figs. S3, S4) showed no obvious change, indicating its tough and flexible properties.

**Table 1:** Mechanical properties of BC films with different wet-stretching

Samples	Strain at the break, %	s.d.	Young’s modulus, GPa	s.d.	Tensile strength, MPa	s.d.	Toughness, MJ/m^−3^	s.d.
BC-00	3.7	0.075	9.2	1.5	116.5	8.0	2.0	0.6
BC-20	3.8	0.05	12.3	1.0	229.9	6.0	4.0	0.3
BC-30	4.0	0.06	41.1	0.9	678.5	6.6	12.7	0.4
BC-40	4.2	0.075	43.6	0.7	1018.8	5.3	20.2	0.4

s*.d.* stands for standard deviation

Additional file: As a rule, all supplementary files are to be referred as additional files. Thus, "Supplementary information" was changed to "Additional file 1". Moreover, titles inside the additional files were also amended to correspond with their modified citations. Please check and advise if action taken is appropriateThank you so much. The modification is ok.

### Electrical conductivity of AgNW@BC film

**Fig. 4:**
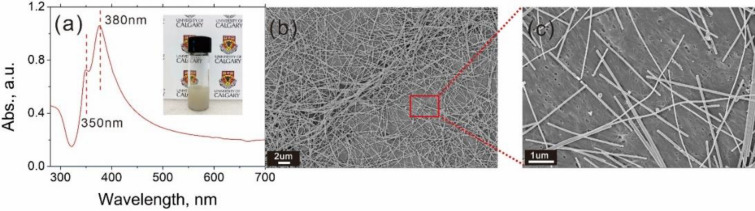
**a** The UV absorbance of AgNW solution; **b–c** SEM images of silver nanowires on BC-40 at **b** lower and **c** higher magnifications

Silver nanowires (AgNW) were synthesized by a polyol process which was based on the reduction of an inorganic salt by a polyol at an elevated temperature. In this method, EG was used as both solvent and reducing agent, PVP was utilized as stabilizing agent, and AgNO_3_ was used as Ag source. According to the UV–vis absorption spectrum of the AgNWs solution (Fig. 4a), there were two narrow peaks at 380 nm and 350 nm. According to previous studies (Yang et al. 2013; Zhang et al. 2019; Tang et al. 2009), the existence of the absorption peaks at 350/380 and 410 nm in the UV–vis spectrum is the characteristic peaks of long AgNWs and silver nanoparticles, respectively. Hence, the absence of the absorption peak at 410 nm in Fig. 4a also verified the high purity of the prepared AgNWs. The UV spectra were unanimous with the SEM images (shown in Fig. 4b–c) which display the size of AgNW. It is noticeable that there were mainly AgNWs with an average length of 2–3 um, besides very few silver nanoparticles observable, showing the high purity of AgNWs. In addition, there is an aggregation of the AgNWs as shown in Fig. 4b probably due to the overlap of the AgNW solution droplets.

**Fig. 5:**
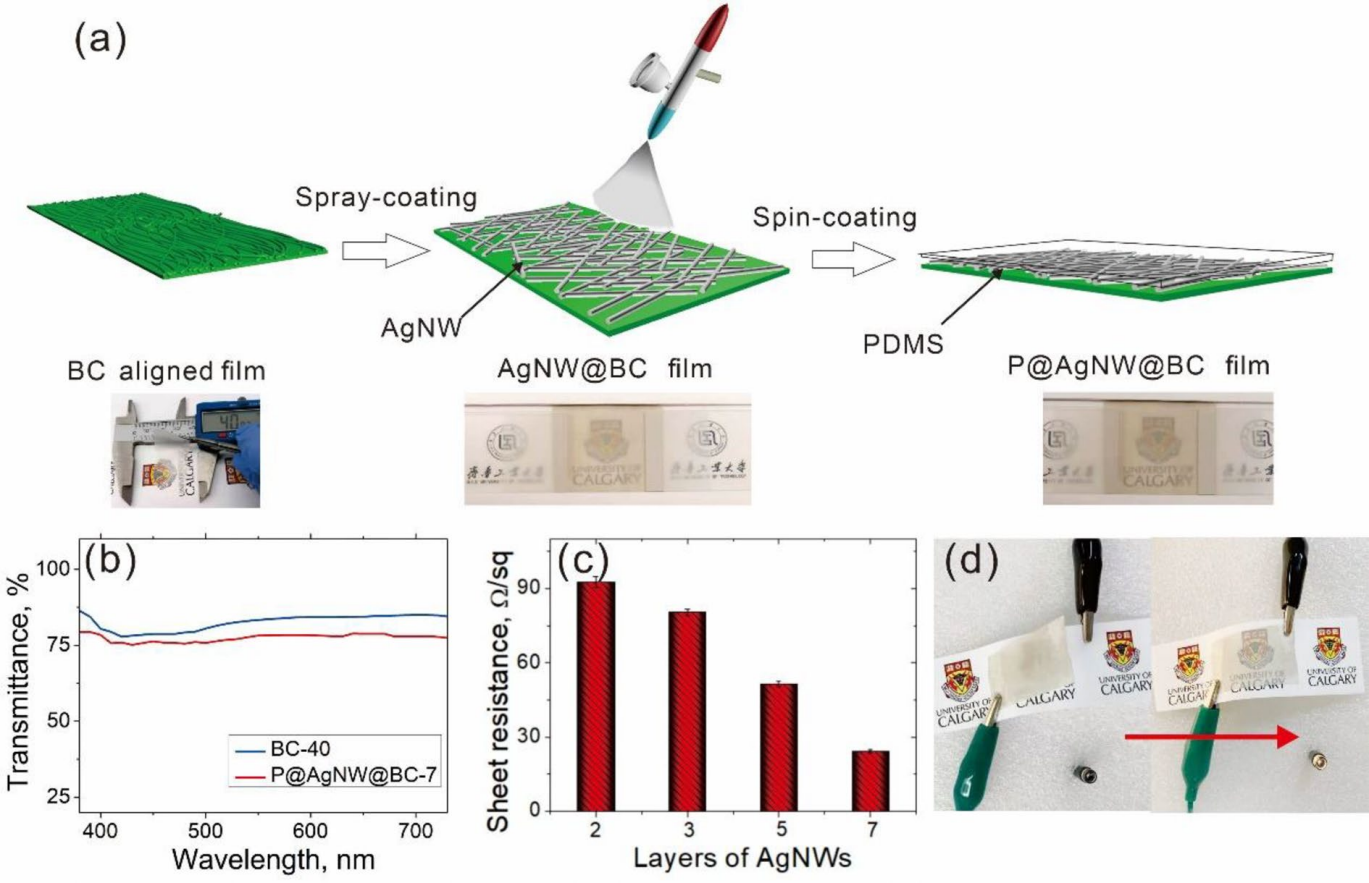
**a** Schematic illustration of the fabrication process of P@AgNW@BC composite film; **b** transparent of the BC-40 film and P@AgNW@BC-7 film; **c** the square resistance of P@AgNW@BC-7 film with different spray-coating layers; **d** photographs of series-wound white light-emitting diodes (LEDs) and conductive P@AgNW@BC-7 film

The BC-40 film was spray-coated with AgNWs and dried in atmosphere. Then spin-coated with PDMS to form P@ AgNW@BC composite film as schematically depicted in Fig. 5a. The BC-40 film showed transparency, whereas, P@AgNW@BC-7 had very small change detectable by the naked eye, as noted by the photography of University Logo covered with P@AgNW@BC-7 (Fig. 5a). Figure 5b reveals that BC-40 film poses excellent transparency with transmittance values of 88% at 550 nm and 89% at 700 nm in the UV–Vis region. However, P@AgNW@BC-7 film has a slight diminution, but it is still very acceptable, with transparency of 79% at 550 nm and 80% at 700 nm, respectively. AgNW solution are not completely transparent and shows absorption in the visible region (400–780 nm) (shown in Fig. 4a), thus the AgNWs layers spray-coated on the BC film lead to the decrease transparency of P@AgNW@BC-7.The electrical properties of P@AgNW@BC film were optimized by adjusting the AgNW layers. When the number of AgNW layers increased, the sheet resistance significantly reduced from 92 to 51 Ω/sq. A minimum sheet resistance value of 23 Ω/sq was observed for the seven-layered AgNWs (P@AgNW@BC-7). Figure 5d presents the luminance of a white LED, in a series circuit with the P@AgNW@BC at a power supply of 3 V. The white LED is lighted as the P@AgNW@BC-7 film attached.

### Joule heating performance of P@AgNW@BC film

**Fig. 6:**
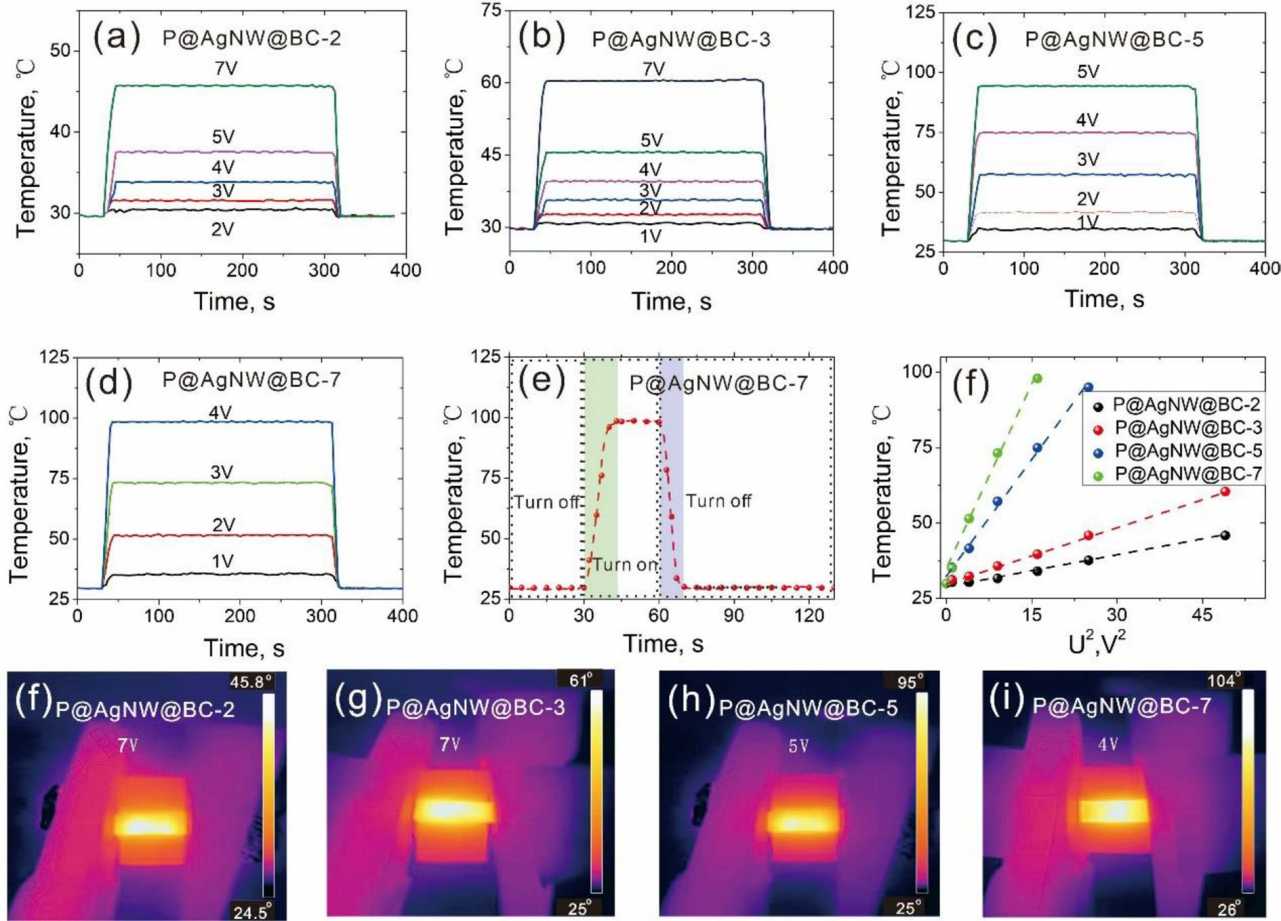
**a–d** Time-dependent temperature change of the P@AgNW@BC composite film with different AgNW spray-coating layers under different applied voltages; **e** the heating and cooling rates of P@AgNW@BC-7 film heater at 4 V; **f–i** infrared images of P@AgNW@BC film heaters with various driving voltages

A high level of electrical conductivity is crucial during the design of electric heaters in order to ensure good heating performance. The time-dependent temperature changes of P@AgNW@BC samples were determined under different voltages on the range of 1–7 V (Fig. 6a–d). The saturation temperatures in all P@AgNW@BC samples increased with the rise of the driving voltage. For P@AgNW@BC-2, the saturation temperature rose from 30 ℃ to 45 ℃ as the driving voltage increased from 1 to 7 V (Fig. 6a). The P@ AgNW@BC sample with high AgNW content was easy to achieve the high saturation temperature at the same driving voltage and required to obtain a similar saturation temperature at a low driving voltage. A saturation temperature of 40 ℃ was obtained at 4 V for P@ AgNW@BC-3, whereas higher saturation temperatures of 75 ℃ and 98 ℃ were achieved at 4 V for P@AgNW@BC-5 and P@ AgNW@BC-7, respectively (Fig. 6b–d). Besides a saturation temperature of 45 ℃ was gained at 7 V for P@ AgNW@BC-2, while a saturation temperature of 57 ℃ is reached at only 3 V for P@ AgNW@BC-5. Moreover, a saturation temperature of 51 ℃ was achieved at an even lower voltage of 2 V for P@AgNW@BC-5. It was interesting to observe that, regardless of the applied voltage, our P@AgNW@BC samples respond very quickly to heating-up processes. As shown in Fig. 6e, when the voltage was applied to the P@ AgNW@BC-7 composite film, its temperature increased rapidly and then reached a saturated value within 13 s. Once the input voltage was switched off, the films cooled down quickly. The heating stability and repeatability of a heater are also important for its practical applications. As shown in Additional file 1: Fig. S5, cyclic heating/cooling was conducted on P@AgNW@BC-7 film heater with on/off switch of driving voltage. The surface temperature steadily increased and decreased during repeated driving voltage for > 200 cycles and the maximum value of the surface temperature was basically the same as the driving voltage both at 2 V and 4V, which indicated the good heating stability and repeatability of P@AgNW@BC-7 film. Figure 6f shows the saturation temperature of the P@ AgNW@BC films corresponding to different drive voltages. According to the energy balance principle, the time-dependent thermal model is developed as (Wang et al. 2020):

$$T={T}_{0}+\frac{{U}^{2}}{RhA}\left(1-{e}^{-\left(\frac{hA}{mc}\right)}\right)t,$$

where *U* stands for driving voltage, *R* is the resistance of the electrical heater, m represents the mass, A is the area, *c* is the specific heat capacity, *h* stands for convective heat-transfer coefficient, *T* stands for the temperature of the electrical heater and T_0_ is the initial ambient temperature. The saturation temperatures of the P@AgNW@BC film heaters (Fig. 6f) showed a highly correlated linear relationship with the square of the applied voltage (*U*^2^), confirming the precision of the theoretical prediction model of saturation temperature at different applied voltages. With constant driving voltage, the heaters with higher AgNW content showed higher saturation temperatures due to reduced electrical resistance. The above results together with the specific switchable characteristics demonstrate that the heating performance of the heaters could be easily adjusted by controlling the AgNW contents or driving the voltage. Furthermore, The Joule heating performances of the P@AgNW@BC film heaters were investigated at various driving voltages, and the temperature was monitored using an FTIR camera as shown in Fig. 6f–i. Obviously, the thermal images present a uniform temperature distribution, which is regarded as an important criterion in electrical heaters.

**Fig. 7:**
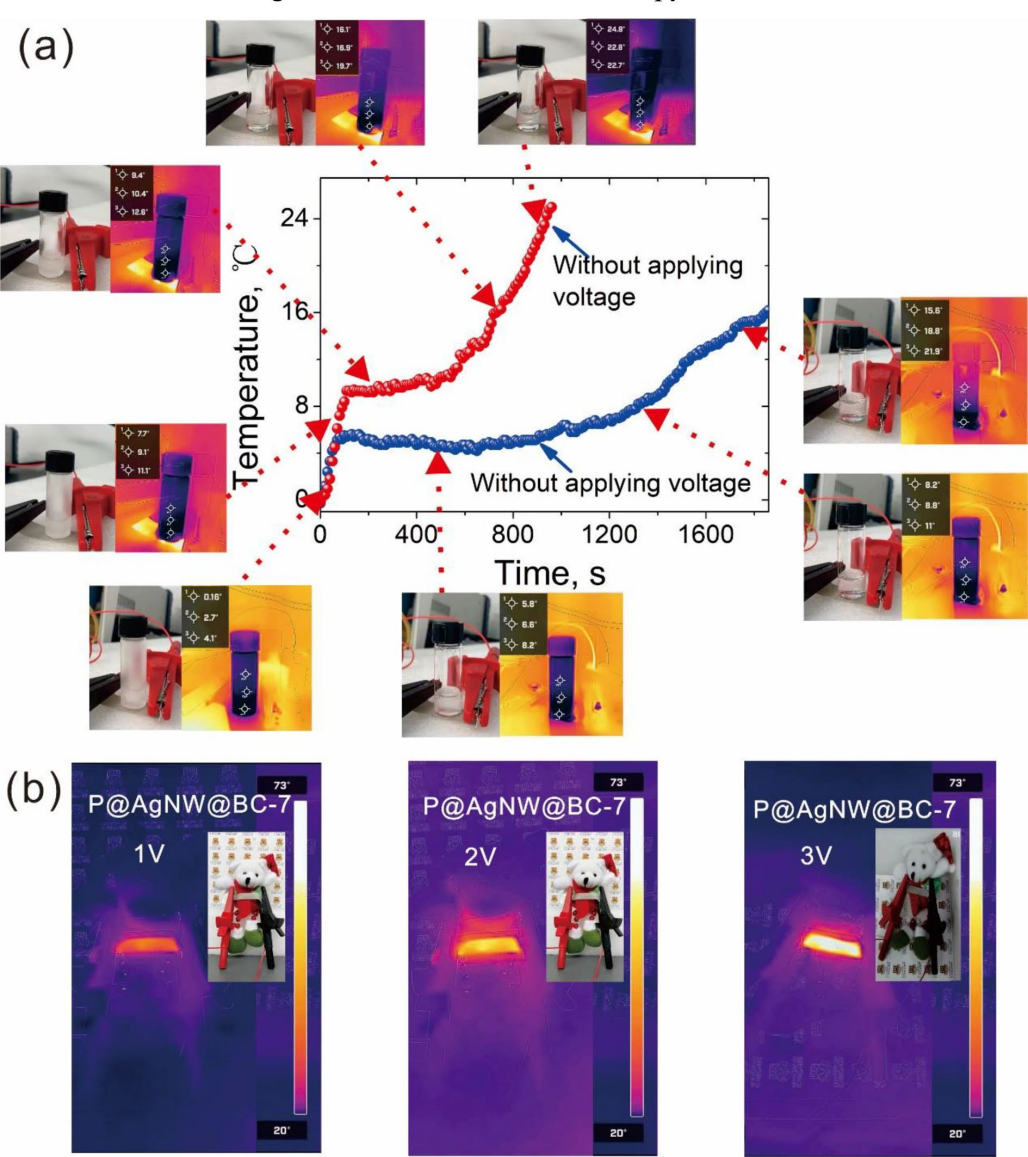
**a** The photographs of deicing device prepared with P@AgNW@BC-7; **b** IR thermal images of P@AgNW@BC-7 wearable thermotherapy at driving voltage of 1 V, 2 V, and 3 V

The low driving voltage ensured the safety of the devices for the human body, while the fast thermal response made the devices reach the desired temperature rapidly. And the wide temperature range endowed the devices with versatile applications. Based on the superior Joule heating performance and high stability, an electric deicing device and a wearable heater had been fabricated by using the P@AgNW@BC-7 film. A simple deicing device (Fig. 7a) was fabricated with a 10 mm width and 40 mm length, which was driven at 4 V. An ice cube (about 1.0 g water) in a glass bottle melted quickly and transferred completely to the liquid phase within 540 s at 3.0 V. During this process, the temperature of the bottom water increased from original − 0.5 ℃ to 8 ℃. More importantly, the water temperature increased to about 25 ℃ via electric heating for another 200 s. As a control, the ice cube without electric heating melted slowly and did not melt completely until 800 s. Figure 7b shows the application of P@AgNW@BC-7 film as an electrical hater in wearable thermotherapy. The P@AgNW@BC-7 film affixed to the chest of a doll was heated up by using different driving voltages from ambient temperature to 35 ℃, 51 ℃, and 73 ℃. This observation shows the great applicability of the developed film in-medical thermotherapy to effectively relieve pain and stiffness within the temperature range of 30–77 ℃. Therefore, the P@AgNW@BC-7 films could successfully be used to construct low-voltage driving devices, such as deicing devices, wearable thermotherapy, and so on.

### The antibacterial properties of P@AgNW@BC film

**Fig. 8:**
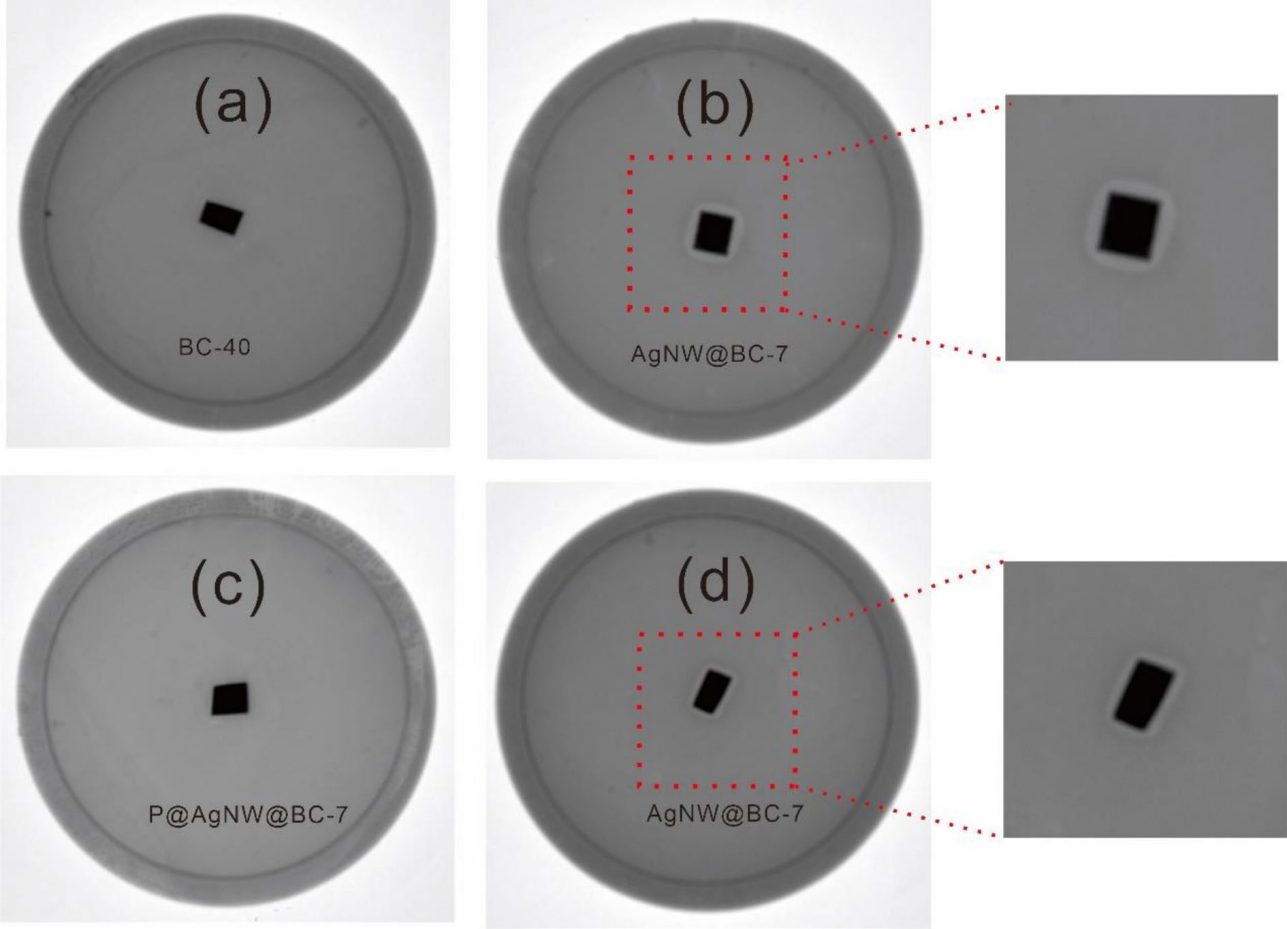
Photographs of *E. coli* cultivated on agar plates for **a** BC-40 film, **b** AgNW@BC-7 film, **c** P@ AgNW@ BC-7 film, **d** AgNW@ BC-7 film stored for 1 year

The antibacterial activity is imperative for the commercialization of thermal management film in many applications specifically those that are related to humans. It has been shown that silver nanowires pose excellent antibacterial properties (Zhou et al. 2021). To explore the antibacterial properties of different prepared films, the antibacterial activities of BC-40, AgNW@BC-7 (AgNW@BC film with 7 AgNW layers), and P@ AgNW@BC-7 films were tested against *E. coli* (ATCC 8739), as shown in Fig. 8. The pristine BC-40 film did not show antibacterial activity (Fig. 8a), whereas the AgNW@BC-7 film yielded good antibacterial performance against *E. coli* (Fig. 8b). This performance originated from the presence of AgNW in the BC film due to the antibiotic activity of AgNW. However, the mechanisms of the antibiotic activity of silver nanomaterials (silver nanowire and silver nanoparticle) are not well established to date and is still a topic of hot debate. Several mechanisms have been proposed (Li et al. 2010; Singh et al. 2016; Vanlalveni et al. 2021). For example, silver nanomaterials bind strongly with phosphorus and sulfur of the extracellular and intracellular membrane proteins, thus affects the cell respiration, replication and the lifetime of the cell. Besides, the interaction between the positively charged Ag ion with the negatively charged cell membranes led to the disruption of the cell morphology and the cell leakage leading to cell death. Apart from that, silver nanomaterials can also bind with the thiol and amino groups of membrane protein resulting in the formation of reactive oxygen species, which inhibits the cell respiration. It has also been suggested that the interaction of silver nanomaterials with cell wall increases the membrane permeability of forming pores or pits and thereby causing the death of bacteria (Morones et al. 2005; Yu and Yam 2004;). As our expected, the P@ AgNW@BC-7 film does not show antibacterial activity (Fig. 8c). The possible reason is that the PDMS covered the AgNW surface of P@AgNW@BC-7 film prohibited the oligodynamic effect of Ag. In addition, it is interesting to note that after one year of storage in the paper envelope placed in ambient environment, the AgNW@BC-7 film still showed excellent antibacterial performance (Fig. 8d).

## Conclusions

In summary, we reported the fabrication P@AgNW@BC film heater with ultrahigh mechanical properties, flexibility, and excellent Joule heating performance. In detail, the aligned BC film was produced and used as a substrate to spray-coated with AgNWs and then spin-coated with PDMS to fabricate the P@AgNW@BC film heater. Wet-stretching and hot-press drying were used to prepare the aligned BC film with cellulose nanofibers. The mechanical properties of the aligned BC film substantially increased with the increase of wet-stretching. The BC-40 film with 40% wet-stretching exhibited ultrahigh strength of 1018 MPa and excellent toughness of 20 MJ m^−3^. In addition, the aligned BC film showed great flexibility that could be shaped to any desired shape. The P@AgNW@BC film heater generated a hot saturation temperature of up to 98 ℃ at 4 V and exhibited a shorter response time (13 s) and long-term stability of saturation temperature. In addition, the AgNW@BC film showed outstanding antibacterial properties even after 1 year of storage. The P@ AgNW@BC film heater with such an excellent performance can be used as portable thermal management such as deicing, defogging, defrosting devices, wearable thermotherapy, etc.

### Supplementary Information

**Additional file 1:** **Fig. S1. **FTIR spectra of BC films with different wet-stretching: **a** spectra from 550 to 4000 cm^-1^ **b** spectra from 550 to 1500 cm^-1^. **Fig. S2.** The density of BC films with different wet-stretching. **Fig. S3.** The schematic diagram of the BC film fold and unfold. **Fig. S4.** The tensile strength of BC films after folding 100 times (BC-00-F, BC-20-F, BC-30-F, and BC-40-F represent the BC-00, BC-20, BC-30, and BC-40 after folding 100 times). **Fig. S5.** Heating stability and repeatability of the P@AgNW@BC-7 film heater upon repeated driving voltages.

## Data Availability

Will be provided based on request.
