# Supplementary material for: Super-strong and high-performance electrical film heater derived from silver nanowire/aligned bacterial cellulose film
Source: Bioresour Bioprocess. 2023 Aug 23;10(1):54. doi: 10.1186/s40643-023-00669-w (PMC10992140; doi:10.1186/s40643-023-00669-w)
Supplement: Supplementary file 1 — Additional file 1: Fig. S1. FTIR spectra of BC films with different wet-stretching: a spectra from 550 to 4000 cm-1 b spectra from 550 to 1500 cm-1. Fig. S2. The density of BC films with different wet-stretching. Fig. S3. The schematic diagram of the BC film fold and unfold. Fig. S4. The tensile strength of BC films after folding 100 times (BC-00-F, BC-20-F, BC-30-F, and BC-40-F represent the BC-00, BC-20, BC-30, and BC-40 after folding 100 times). Fig. S5. Heating stability and repeatability of the P@AgNW@BC-7 film heater upon repeated driving voltages. [file 40643_2023_669_MOESM1_ESM.docx]

**Supporting Information**

Super-Strong and High-Performance Electrical Film Heater Derived from Silver Nanowire/Aligned Bacterial Cellulose Film

Guichun Hu ^1,2^, Amir Varamesh^1^, Na Zhong^1^, Fangong Kong^2^, and Jinguang Hu^1^*

^1^ Department of Chemical & Petroleum Engineering, Schulich School of Engineering, Calgary, Alberta, T2N 1N4, Canada;

^2^ State Key Laboratory of Biobased Material and Green Papermaking, Faculty of Light Industry, Qilu University of Technology, Shandong Academy of Sciences, Jinan 250353, P.R. China;

**Experimental**

**Characterization**

PerkinElmer FT-IR spectrometer Frontier (Frontier, Perkin Elmer, USA) were utilized to estimate the surface functional groups of different samples in the range of 4000–550 cm^-1^ at 64 scans with an average scanning rate of 2 cm^-1^. The density of the BC film was determined by dividing the sample weight by the sample volume as measured by a digital caliper.

**Results and Discussion**


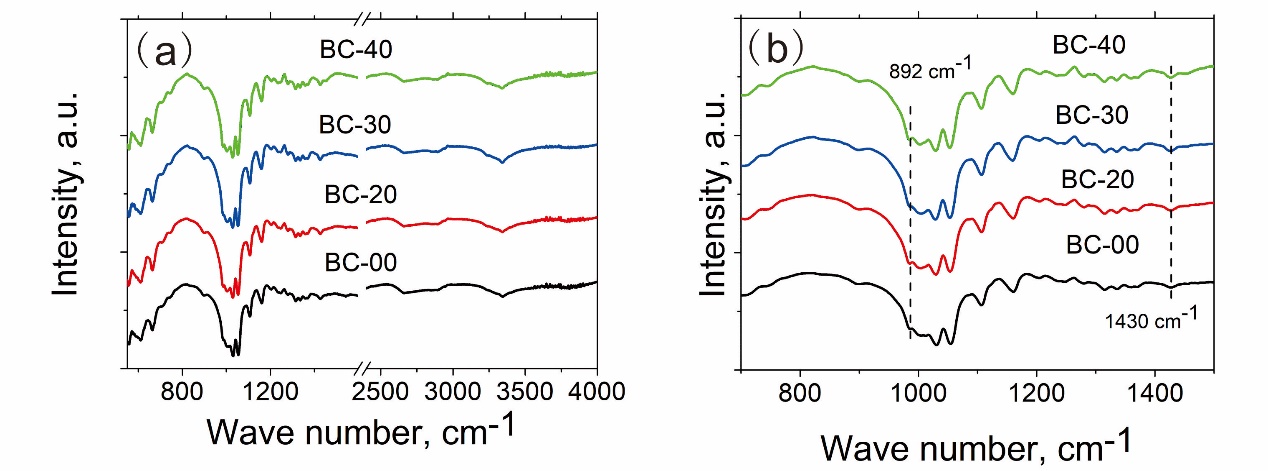


**Fig. S1** FTIR spectra of BC films with different wet-stretching: (a) spectra from 550 to 4000 cm^-1^ (b) spectra from 550 to 1500 cm^-1^.





Fig. S2 The density of BC films with different wet-stretching.


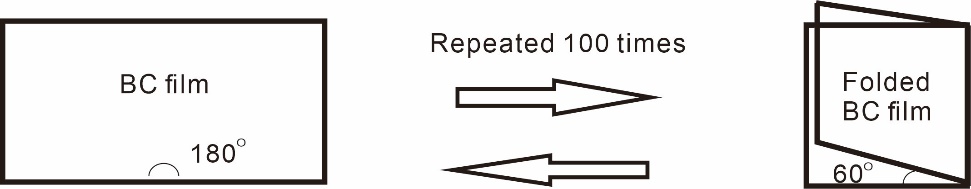


Fig. S3 The schematic diagram of the BC film fold and unfold.





Fig. S4 The tensile strength of BC films after folding 100 times (BC-00-F, BC-20-F, BC-30-F, and BC-40-F represent the BC-00, BC-20, BC-30, and BC-40 after folding 100 times).


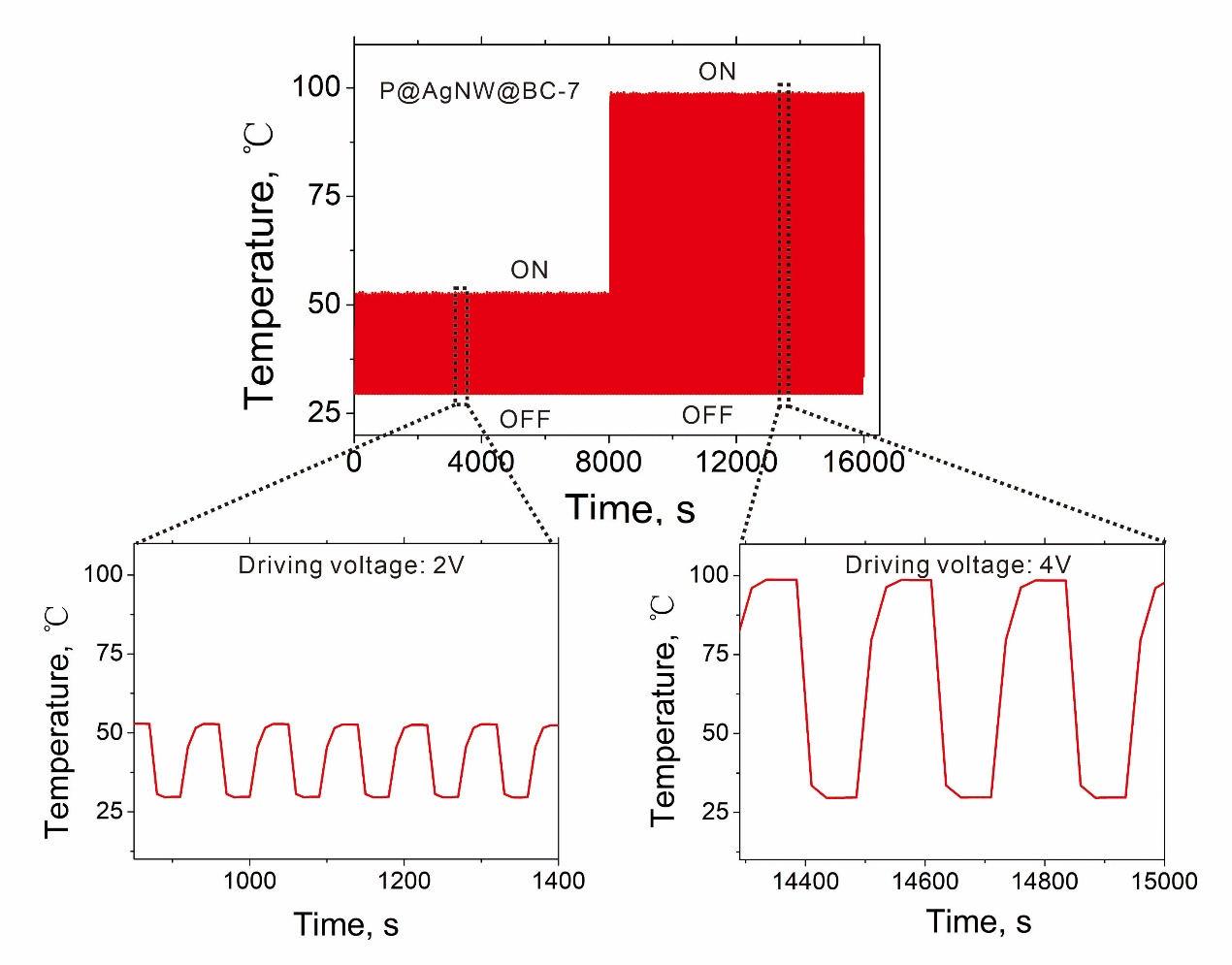


Fig. S5 Heating stability and repeatability of the P@AgNW@BC-7 film heater upon repeated driving voltages.
